# Work-related psychosocial challenges and coping strategies among nursing workforce during the COVID-19 pandemic: a scoping review

**DOI:** 10.1186/s12912-023-01368-9

**Published:** 2023-06-19

**Authors:** Merri Iddrisu, Collins Atta Poku, Eva Mensah, Priscilla Y. A. Attafuah, Gladys Dzansi, Samuel Adjorlolo

**Affiliations:** 1 School of Nursing and Midwifery, University of Ghana, Accra, Ghana; 2 Department of Nursing, Kwame Nkrumah University of Science and Technology, Kumasi, Ghana

**Keywords:** Coping strategies, COVID-19, Nurses, Psychosocial challenges, Work-related

## Abstract

**Background:**

Nurses and midwives have been stretched by the COVID-19 pandemic amidst the heroic roles they have played during the peak of the COVID-19 pandemic. Nurses stood tall among their peers in the healthcare industry saving lives. The pandemic has had a toll on nurses physically, psychologically, and socio-economically. The numerous deaths have traumatized nurses coupled with the fear of possible infection. Nurses have seen their colleagues and loved ones lose their lives to the pandemic, nevertheless, they still render care to patients no matter the circumstances. Due to that, it is imperative to ascertain the extent to which nurses who are much needed in healthcare delivery have been affected by this pandemic. This scoping review used Arksey and O’Malley’s review methodology to investigate the nature of work-related psychosocial challenges nurses encountered during the peak of the pandemic, noting the major contributors to the challenges and the coping strategies used to address them.

**Methods:**

We performed a scoping review and searched for articles from five databases including PUBMED, CINAHL, SCOPUS, Google Scholar, and Grey literature from December 2019 to December 2021. A total of 7,334 articles were retrieved for the study but 45 met the inclusion criteria.

**Results:**

Work-related psychosocial challenges identified included stress, burnout (emotional exhaustion and depersonalization), Post-Traumatic Stress Disorder, depression, sleeplessness, and anxiety. Factors that accounted for the challenges were inadequate personal protective equipment (PPEs), discomfort using the PPEs, extreme workload, and fatigue. Nurses experienced job insecurity, business closure, and separation from family and loved ones, and these contributed to their challenges. Strategies used to deal with the challenges centred on emotion-focused and problem-focused coping.

**Conclusions:**

The study recommends regular counselling and support for all nurses working at the frontline to help them better cope with the devastating effects of the pandemic so that they could build resilience towards future pandemics.

## Introduction

The nursing workforce forms more than 60% of the healthcare workforce and it is reported to be the backbone of the healthcare industry, therefore their safety and welfare should be a priority for all players in the health sector [1]. Nurses are found everywhere, in the communities and health facilities both rural and urban delivering care. During the peak of the COVID-19 pandemic, nurses from all sectors of the profession played various roles which have resulted in the successes seen in the fight against the pandemic. While public health nurses and community health nurses were doing contact tracing and follow-up of cases [2], mainstream clinical nurses were found at the emergency units and isolation centres handling suspected and confirmed cases [3]. Critical care nurses were found in the intensive care units (ICUs) helping to revive those with respiratory distress, and the seriously ill assisted in feeding and taking care of their hygiene needs [4]. Furthermore, nurses handled dead bodies before their conveyance to the mortuary and gave psychosocial support to family members [5].

The roles nurses continue to play in combating the COVID-19 pandemic are enormous. The COVID-19 outbreak has strained the global healthcare system, with conditions getting worse as a result of overcrowding and caring for several patients in the hospital with proven or suspected COVID-19 infections and a lack of supplies, beds, and the workforce. Nurses have been confronted with numerous work-related challenges ranging from physical (injury/musculoskeletal problems) to psychosocial (stigma/verbal abuse) [6, 7]. These challenges have resulted in many nurses developing mental health disorders, quitting the profession, and some losing their lives [8, 9]. For these reasons, international nursing organisations, for example, the International Council of Nurses and the American Nurses Organisation have issued statements and envisaged that the future of the nursing profession is gloomy if nothing is done about the situation [10].

Previous pandemics caused mental health decline in people; however, the COVID-19 pandemic came with lockdowns, the mandatory wearing of PPEs, and movement restrictions in many countries that compounded the problem. Though there were limitations in healthcare, such limitations and stringent observation of protocols in healthcare have been incomparable globally due to differences in the availability of resources. Though there were issues with PPEs availability and space for patients, nurses in less endowed countries and health facilities suffered more. Hence, there were worries that nurses experienced anxiety due to concerns about the possibility of getting infected with COVID-19 [11].

The demanding working conditions nurses found themselves in made them develop serious psychological issues like insomnia, depression, and anxiety. In addition to the stress brought on by their core duties as nurses, their mental health got worse as a result of restrictions on their daily activities including bans on going out in public. Therefore, identifying risk and resilience characteristics linked to mental health issues related to COVID-19 is essential. This could assist those at risk and boost their resilience. COVID-19 did not only affect nurses’ emotions, but it also changed the way they cope [2].

It is therefore critical to ascertain stress management strategies that can aid nurses in coping with the extraordinarily trying conditions brought on by COVID-19 as well as for use in similar pandemics. Techniques for coping with stress and other psychosocial challenges during the 2002 SARS outbreak have been shown to enhance psychological symptoms and overall health. Further studies have revealed that during previous pandemics, nurses were able to handle the situation by controlling their emotions and adjusting to them. Therefore, using coping mechanisms under pressure may prevent a mental health catastrophe. Additionally, suggesting coping mechanisms for healthcare professionals will be crucial in reducing the detrimental impacts of COVID-19 [12].

Instituting holistic psychosocial support will help nurses continuously build resilience, thereby improving a safe working environment to promote quality of life, increase work output, and push towards the achievement of the Sustainable Development Goals (SDGs) and the Universal Health Coverage (UHC) by 2030 [13].

This review, therefore, sought to assess the various work-related psychosocial challenges nurses encountered during the pandemic, noting the major contributors to the challenges and the coping strategies nurses adopted to deal with them.

## Methods

### Design

A scoping review was performed as it aims at determining the range and extent of research activity, appraising key research outcomes, and finding gaps in the literature. A scoping review is usually undertaken to establish the need for a systematic review and to guide future research [14]. Scoping review approach was used because the researchers wanted to assess the extent of the body of literature on work-related psychosocial challenges of nurses associated with COVID-19, and the coping strategies. Investigating such a topic calls for an exploratory yet thorough mapping of essential concepts, evidence, and research gaps which is ideally suited for scoping review method [15–17]. A study protocol was developed but this was not published in the public domain. In this study, the six [6] stages of Arksey and O’Malley’s scoping review methodology were followed [15]: [1] specifying the research goals and identifying research questions, [2] establishing the inclusion and exclusion criteria for the search, [3] identifying the search strategies, [4] charting the results, [5] discussing the results, and [6] providing conclusions and recommendations were followed. For the current review, details of the six stages are provided as follows:

### Stage 1: specifying goals of the research and identifying research questions

The following questions guided the review:

1. What work-related psychosocial challenges do nurses encounter in the COVID-19 pandemic?
2. What coping strategies do nurses use to deal with work-related psychosocial challenges in the COVID-19 pandemic?

The study results were clearly and comprehensibly summarised. Through an iterative process of charting and analysing the data, extra pertinent result associated with the work-related psychosocial challenge was identified and presented below:

3. What factors account for the work-related psychosocial challenges among nurses?

### Stage 2: inclusion and exclusion criteria

From each database, only peer-reviewed literature published from December 2019 to December 2021 was searched. In addition to the peer-reviewed literature, grey literature published within the specified period mentioned earlier was considered. The inclusion and exclusion criteria are presented in Table 1.

i. Population: nurses involved in the care of COVID-19 patients in the clinical setting.
ii. Exposure: working with COVID-19 cases, either confirmed or suspected.
iii. Intervention: coping strategies that helped to reduce or prevent work-related challenges.
iv. Outcome: psychosocial challenges that resulted from the COVID-19 pandemic.
v. Study design: grey literature and peer-review publications comprising qualitative, quantitative, and mixed-method designs were included in the review (primary studies/ original articles).

**Table 1:** A comprehensive description of the eligibility of articles

Criterion	Inclusion	Exclusion
**Time frame**	Articles published from December 2019 to December 2021	All related articles before December 2019 and those after December 2021
**Language**	All languages/Full article	Nil
**Participants**	All categories of nurses	Other healthcare professionals, physicians, and students
**Place**	All countries	Nil
**Type of source**	Original research published in peer-reviewed journals and grey literature	Books, commentaries, newspapers, unpublished articles
**Settings**	Hospitals and other healthcare facilities	Nursing homes and schools
**Interesting phenomena**	All work-related challenges and coping strategies nurses face	Studies not related to the study focus

### Stage 3: identifying relevant studies

The search was conducted in the following electronic databases: PUBMED, CINAHL, SCOPUS, and Google Scholar for articles published between December 2019 and December 2021. To guide the review, the PCC tool was used to separate the concepts: [1] Population/Problem (Nursing Workforce); [2] Concept (Work-Related Psychosocial Challenges and Coping Strategies); [3] Context (during the COVID-19). The keywords included challenges, nurses, pandemic, COVID-19, SAR-CoV2, work-related, and coping strategies. The search string (Nurs* OR Midwi*) AND (“Work-related” OR occupational OR “job-related”) AND (challenges OR problems) AND (“COVID-19” OR “SARS-COV-2”) AND (“Coping-strateg’’ OR Resilience) was compiled and first used for PUBMED and later adapted for the other databases. In addition to the search string, the following MESH terms were employed: nurse, nurse clinician, midwife, midwives, and midwifery, which were used to search for the population. The concept of work-related psychosocial challenges and coping strategies were searched using MeSH terms separated by BOOLEAN operators AND/OR: job-related OR occupational OR work OR professional OR work-related OR hospital-related AND psychosocial AND challenges OR factors OR problems OR coping strategies OR coping behaviour OR resilience. The context was searched using COVID OR Covid-19 OR COVID-19 OR SARS-COV-2, as MeSH terms. All remaining terms were free terms. Google Scholar was also searched using the terms nurse AND work-related challenges AND coping strategies in SARS-COV2 OR COVID-19 pandemic. The references to the included articles were also hand-searched to see if any studies were missed in the initial search. The following grey literature sources were also searched: MedNar, Open Grey, and Trove. Articles were considered for the analysis if they reported both or separate results for work-related challenges and coping strategies among nurses.

The title, the abstract, and the full text of the articles were independently assessed by the three reviewers. On the occasion of differences in opinion, an agreement was reached through discussion and consensus. Stage 4 is detailed in the results section of this study followed by stages 5 and 6 in the discussion and conclusion segments respectively.

## Results

### Data charting process and synthesis

**Fig. 1:**
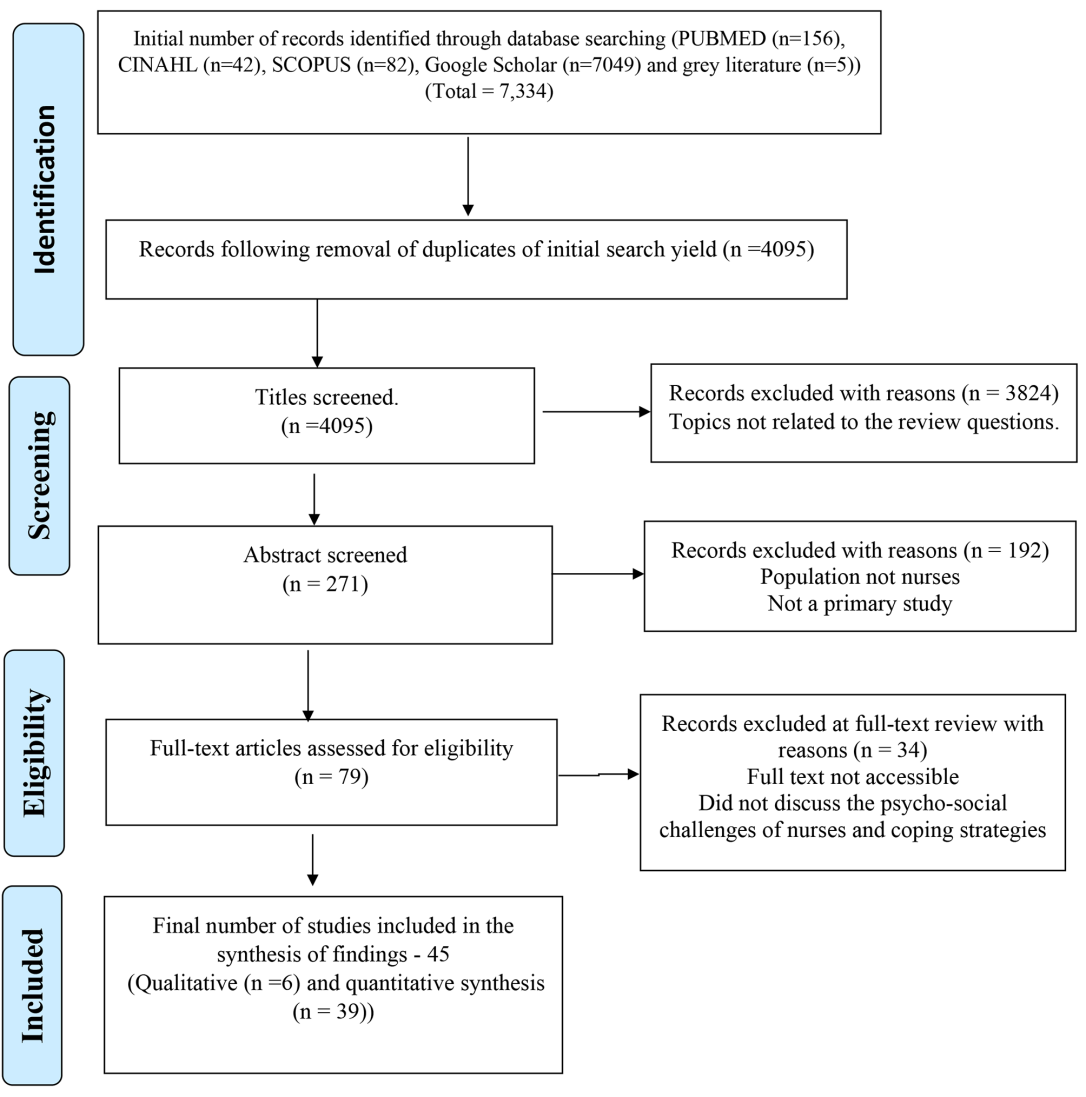
PRISMA ScR Flow Diagram depicting the study selection process

The results from the selected databases generated 7,334 articles. Articles with abstracts were exported using Zotero software (5.0.96), and data were entered into a standardized data chart using Microsoft Word. Information on author(s), year of publication, title, study purpose, study location, study design, and findings were recorded. After duplicate articles were removed, two reviewers critically read the abstracts of 271 articles. An additional 192 articles were then excluded after reviewing the abstracts as they did not meet the inclusion criteria of the population being nurses and primary articles. In all, 79 articles related to the research questions were included in the review but only 45 fully met the inclusion criteria of attaining their full text and also discussing the psychosocial challenges of nurses and/or coping strategies. Figure 1 details the Preferred Reporting Items for Systematic Reviews and Meta-Analyses (PRISMA) flow diagram for selecting the articles. Based on the review aim, further data were extracted to be able to answer the review question. To synthesize the extracted data, we used the review aim as a heuristic guide to formulating codes across the included studies following which similar codes were aggregated to formulate sub-categories. Finally, similar sub-categories were merged to develop categories that formed the basis of undertaking a narrative synthesis.

Out of the 45 studies, there were 39 quantitative studies; 38 cross-sectional designs [18–55], a longitudinal study [56], and six [6] qualitative studies [57–62]. All studies were conducted in a hospital setting, with the sample size for the quantitative studies varying between 91 [24] and 7542 [35]. The qualitative studies had sample sizes of 12 [58], 15 [61], 17 [62], 20 [60], 26 [57], and 55 [59]. Eight [8] of the studies were undertaken in China [26, 45, 51–56], six [6] studies in the United States [18, 23, 47, 48, 59, 63] and two [2] each from Iran [58, 61], Italy [30, 41], Lebanon [19, 20], Philippines [38, 62], Poland [24, 37], South Korea [32, 39], Spain [40, 46], and the UK [43, 44], a study each from Canada [60], Cyprus [29], Ecuador [28], Egypt [49], Germany [35], Indonesia [42], Israel [31], Jordan [57], Malaysia [25], Norway [50], Saudi Arabia [22], South Africa [27], and three multi-country studies [33, 34, 36] as presented in Table 2.

**Table 2:** Characteristics of Included Studies

No	Author/ Year	Title	Purpose	Origin	Sample	Design
1	Mo et al., 2020	Work stress among Chinese nurses to support Wuhan in fighting against the COVID-19 epidemic	To investigate the work stress among Chinese nurses supporting Wuhan in fighting against COVID-19 infection and the relevant influencing factors.	China	180	Cross-sectional
2	Sagherian et al., 2020	Insomnia, fatigue and psychosocial well-being during COVID-19 pandemic: A cross-sectional survey of hospital nursing staff in the United States	To describe the levels of insomnia, fatigue and inter-shift recovery, and psychological well-being (burnout, post-traumatic stress and psychological distress), and examine differences in these measures based on work-related characteristics among nursing staff during the COVID-19 pandemic.	United States	587	Cross-sectional
**3**	Arnetz et al., 2020	Nurses Report of Stressful Situations during the COVID-19 Pandemic: Qualitative Analysis of Survey Responses	To explore perceptions of the most salient sources of stress in the early stages of the coronavirus pandemic in a sample of U.S. nurses.	United States	455	Cross-sectional survey
4	Cai et al., 2020	Nurses endured high risks of psychological problems during the epidemic of COVID-19 in a longitudinal study in Wuhan China	To assess the magnitude of the psychological status and associated risk factors among nurses in the pandemic centre in Wuhan, China.	China	709 (during the outbreak) and 621 (during the stable period)	Longitudinal survey
5	Coffré & Aguirre, 2020	Feelings, Stress, and Adaptation Strategies of Nurses against COVID-19 in Guayaquil	To explore the feelings, stress factors, and adaptation strategies of nurses during the COVID-19 pandemic	Ecuador	155	A cross-sectional, descriptive study
*6*	Galletta et al., 2021	Worries, Preparedness, and Perceived Impact of Covid-19 Pandemic on Nurses’ Mental Health	To analyse how risk factors such as perceived impact, preparedness for the pandemic, and worries associated with mental health outcomes (crying, rumination and stress) in nurses.	Italy	894	Cross-sectional study design
7	Marthoenis et al., 2021	Investigating the burden of mental distress among nurses at a provincial COVID-19 referral hospital in Indonesia: a cross-sectional study	To assess the burden of depression, anxiety, and stress, and explore if socio-demographic factors affect mental distress variables among nurses working at the emergence of the COVID-19 pandemic in Indonesia	Indonesia	491	Cross-sectional survey
8	Poortaghi et al., 2021	Exploring nursing managers’ Perceptions of nursing workforce management during the Outbreak of COVID-19: a content analysis study	To explore nurse managers’ perception of workforce management during the COVID-19 pandemic	Iran	15	Descriptive qualitative design
*9*	Sadang, 2021	The lived experience of Filipino Nurses’ Work in COVID-19 Quarantine Facilities: A Descriptive Phenomenological Study	To explore and describe the meaning of nurses’ work in the community quarantine facilities of Lanao del Sur Province amidst the COVID-19 pandemic	Philippine	12	Descriptive Phenomenological study
10	Lorente et al., 2021	Nurses´ stressors and psychological distress during the COVID-19 pandemic: The mediating role of coping and Resilience	This study analyses the cross-sectional effect of sources of stress during the peak of the COVID-19 pandemic on nurses´ psychological distress, with a focus on the mediating role of coping strategies, both problem-focused and emotion-focused and resilience.	Spain	421	Cross-sectional study
11	Moradi et al., 2021	Challenges experienced by ICU nurses throughout the provision of care for COVID-19 patients: A qualitative study	To explore the challenges experienced by ICU nurses throughout the provision of care for COVID-19 patients	Iran	17	Qualitative descriptive
12	Xiong et al., 2020	The Psychological Status and Self-Efficacy of Nurses During COVID-19 Outbreak: A Cross-Sectional Survey	To examine the psychological status and self-efficacy of nurses still working in public hospitals during the COVID-19 outbreak, and explore the relationships among demographic variables, anxiety, depression, and self-efficacy.	China	223	Descriptive cross-sectional survey
13	L. Zhang et al., 2021	Burnout in Nurses during the COVID-19 Pandemic in China: New Challenges for public health	To assess burnout in nurses during the COVID-19 pandemic	China	336	Cross-sectional survey
14	Zheng et al., 2021	Prevalence and associated factors of depression, anxiety, and stress among Hubei paediatric nurses during the COVID-19 pandemic	To evaluate the levels of depression, anxiety, and stress among Hubei paediatric nurses during the COVID-19 pandemic and to analyse the potential factors associated with them	China	614	Cross-sectional study
15	Waage et al., 2021	Sleep patterns among Norwegian nurses between the first and second wave of the COVID-19 pandemic	To investigate sleep patterns among Norwegian nurses, after the first wave, during a period with very low rates of COVID-19.	Norway	1261	Cohort study
16	X. Zhang et al., 2021	Psychological and occupational impact on healthcare workers and its associated factors during the COVID-19 Outbreak in China	To assess the psychological and occupational impact of the COVID-19 outbreak on HCWs and to identify the risk and protective factors contributing to adverse outcomes.	China	946	Survey
17	Zhan et al., 2020	The Current Situation and Influencing Factors of Job Stress Among Frontline Nurses Assisting in Wuhan in Fighting COVID-19	To explore the current situation and influencing factors of job stress among clinical first-line nurses fighting COVID-19.	China	110	Survey
18	McFadden et al., 2021	A Cross-Sectional Examination of the Mental Wellbeing, Coping and Quality of Working Life in Health and Social Care Workers in the UK at Two Time Points of the COVID-19 Pandemic	To compare cross-sectional data collected from health and social care professionals in the UK at two different time points (Phase 1: May–July 2020; Phase 2: Nov 2020–Jan 2021) during the COVID-19 pandemic.	United Kingdom	Phase 1: 3290 Phase 2: 3499 responses	Cross-sectional survey
19	Nowell et al., 2021	A grounded theory of clinical nurses’ process of coping during COVID-19	To explore clinical nurses’ process of coping during COVID-19 and develop a grounded theory that can be used by leaders to support clinical nurses during a disaster.	Canada	20	Grounded Theory
20	Fteropoulli et al., 2021	Beyond the physical risk: Psychosocial impact and coping in healthcare professionals during the COVID-19 pandemic	To examine the psychosocial impact and identify risk factors for poor psychosocial outcomes in healthcare professionals during the Coronavirus disease 2019 (COVID-19) pandemic in Cyprus.	Cyprus	1071	Cross-sectional study
21	AlJhani et al., 2021	Burnout and coping among healthcare providers working in Saudi Arabia during the COVID-19 pandemic	to estimate the frequency and level of burnout and its association with coping strategies among physicians and nurses in Saudi Arabia during the COVID-19 Pandemic	Saudi Arabia	403	Cross-sectional study
22	Molero-Jurado et al., 2021	Coping Strategies as a Mental Health Protection Factor of Spanish Nurses during COVID-19	To analyze the relationships between nurses’ coping strategies and health, with attention to factors related to the perceived threat and/or someone close to them is COVID-19 positive.	Spain	351	Cross-sectional study
23	Cui et al., 2021	Impact of COVID-19 on Anxiety, Stress, and Coping Styles in Nurses in Emergency Departments and Fever Clinics: A Cross-Sectional Survey	To identify the impact of COVID-19 on the psychology of Chinese nurses in emergency departments and fever clinics and to identify associated factors.	China	453	Cross-sectional survey
24	Alameddine et al., 2021	Factors Associated with the Resilience of Nurses during the COVID-19 Pandemic	To determine the level of resilience in the nursing workforce and its relationship to burnout, intention to quit, and perceived COVID-19 risk.	Lebanon	511	Cross-sectional study
25	Htay et al., 2021	How healthcare workers are coping with mental health challenges during the COVID-19 pandemic? - A cross-sectional multi-countries study	To investigate the coping strategies among healthcare workers from different countries and their attitude towards teamwork during the COVID-19 pandemic.	Albania, Egypt, Iraq, Kenya, Mozambique, Myanmar, Palestine, Philippines, South Africa, Tanzania, Uganda, and Zimbabwe	2166	Cross-sectional study
26	Kotrotsiou et al., 2021	Investigating Nurses Stress Response Strategies During the COVID-19 Pandemic	To explore both the stress response strategies of Greek nurses’ parties working in Greece and Europe during the COVID-19 pandemic and the degree of potential correlation between socio-demographic data and response strategies	Greece, the UK and Australia	550	Survey
27	Engelbrecht et al., 2021	Post-Traumatic Stress and Coping Strategies of South African Nurses during the Second Wave of the COVID-19 Pandemic	To investigate post-traumatic stress and coping strategies of nurses during the second wave of COVID-19 in the country	South Africa	286	Cross-sectional survey
28	Hummel et al., 2021	Mental Health Among Medical Professionals During the COVID-19 Pandemic in Eight European Countries: Cross-sectional Survey Study	To compare the mental health of medical professionals with nonmedical professionals in different European countries during the COVID-19 pandemic	Germany, the United Kingdom, Spain, France, Portugal, Austria, Italy, and Switzerland	609	Cross-sectional Study
29	Norman et al., 2021	Moral distress in frontline healthcare workers in the initial epicentre of the COVID-19 pandemic in the United States: Relationship to PTSD symptoms, burnout, and psychosocial functioning	To identify common dimensions of COVID-19 moral distress; and to examine the relationship between moral distress, and positive screening for COVID19‐related posttraumatic stress disorder (PTSD) symptoms, burnout, and work and interpersonal functional difficulties	United States	2579	Survey
30	Ali et al., 2021	Major Stressors and Coping Strategies of Frontline Nursing Staff During the Outbreak of Coronavirus Disease 2020 (COVID-19) in Alabama	To investigate the major stressors and coping strategies reported by nurses working directly with potentially infectious patients in Alabama, United States, during the COVID-19 pandemic.	United States	109	Cross-sectional survey
31	Norful et al., 2021	Primary Drivers and psychological manifestations of Stress in the frontline healthcare workforce during the Initial COVID-19 Outbreak in the United States	To understand the physical and psychological impact of high-stress clinical environments and contributory factors of burnout in the multidisciplinary healthcare workforce during the initial outbreak of COVID-19.	United States	55	Qualitative study
32	Jerg-Bretzke et al., 2021	Psychosocial Impact of the COVID-19 Pandemic on Healthcare Workers and Initial Areas of Action for Intervention and Prevention—The egePan/VOICE Study	To describe and analyze specific areas of workload among different groups of healthcare workers during the first wave of the COVID-19 pandemic.	Germany	7542	Survey
33	Said and El-Shafei, 2020	Occupational Stress, job satisfaction, and Intent to Leave: nurses working on the front lines during the COVID-19 Pandemic in Zagazig City, Egypt	To assess occupational stress, job satisfaction, and intent to leave among nurses dealing with suspected COVID-19 patients	Egypt	420	Cross-sectional study
34	Alameddine et al., 2021	Resilience of nurses at the epicentre of the COVID-19 pandemic in Lebanon	To investigate the level and factors associated with the resilience of nurses practising at the main COVID-19 referral centre in Lebanon.	Lebanon	265	Cross-sectional survey
35	Hamama et al., 2021	Psychological distress and perceived job stressors among hospital nurses and physicians during the COVID-19 outbreak	To examine self-reported job-related stressors induced by the COVID-19 pandemic and psychological distress among hospital nurses and physicians	Israel	172	Cross-sectional design
36	Kowalczuk et al., 2021	Relationships Between Sleep Problems and Stress Coping Strategies Adopted by Nurses Including Socio-Occupational Factors	To investigate relationships between excessive sleepiness and insomnia in interaction with selected socio-occupational factors and stress-coping strategies among nurses	Poland	448	Cross-sectional design
37	Khatatbeh et al., 2021	The Experiences of Nurses and Physicians Caring for COVID-19 Patients: Findings from an Exploratory Phenomenological Study in a High Case-Load Country	To explore the lived experience of physicians and nurses caring for patients with COVID-19 in Jordan.	Jordan	26	Interpretative phenomenology
38	Hong et al., 2021	Resilience and Work-Related Stress May Affect Depressive Symptoms in Nursing Professionals during the COVID-19 Pandemic Era	To investigate the effect of nursing professionals’ resilience on their mental health, work-related stress, and anxiety in response to the COVID-19 pandemic	South Korea	824	Survey
39	Lee et al., 2021	Risk Perception, Unhealthy Behavior, and Anxiety Due to Viral Epidemic Among Healthcare Workers: The Relationships with Depressive and Insomnia Symptoms During COVID-19	To investigate the relationship between mental health problems and unhealthy behaviours among healthcare workers in response to the COVID-19 pandemic	South Korea	406	Survey
40	Betke et al., 2021	Sense of coherence and strategies for coping with stress among nurses	To describe the specific relationship between the sense of coherence and strategies for coping with stress in a group of professionally active nurse	Poland	91	Survey
41	Chui et al., 2021	The COVID-19 Global Pandemic and Its Impact on the Mental Health of Nurses in Malaysia	To assess the impact of psychological distress that COVID-19 has on nurses and their coping strategies.	Malaysia	859	Cross-sectional survey
42	Abuatiq and Borchardt, 2021	The Impact of COVID-19: Nurses’ Occupational Stress and Strategies to Manage It	To explore the occupational stress perception of nurses and how they manage it during the COVID-19 pandemic	United States	236	Cross-sectional survey
43	Marcolongo et al., 2021	The Role of Resilience and Coping among Italian healthcare workers during the COVID-19 Pandemic	To evaluate the psychological state of healthcare workers (HCWs) in the field of rehabilitation during the COVID-19 pandemic	Italy	334	Cross-sectional study
44	McFadden et al., 2021	The Role of Coping in the Wellbeing and Work-Related Quality of Life of UK Health and Social Care Workers during COVID-19	To examine the relationship between coping strategies and well-being and quality of working life in nurses, midwives, allied health professionals, social care workers and social workers who worked in health and social care in the UK during its first wave of COVID-19	United Kingdom	3425	Cross-sectional study
45	Labrague and de los Santos, 2020	Fear of COVID-19, psychological distress, work satisfaction and turnover intention among frontline nurses	To examine the relative influence of fear of COVID-19 on nurses’ psychological distress, work satisfaction and intent to leave their organisation and the profession.	Philippines	261	Cross-sectional research design

### Work-related psychosocial challenges among nurses

The results on psychosocial work-related challenges among nurses and their coping strategies were descriptively summarized. The challenges identified were categorised into psychological and social with a brief description given under each. The factors accounting for the challenges and the coping strategies used have been provided in Table 3.

**Table 3:** Data synthesis

No	Authors/ year/ setting	Key findings	Codes	Sub-categories	Categories
1	Mo et al., 2020 China	• Anxiety and stress were identified among nurses. • Factors that accounted for stress included the nurse being the only child in the family, the severity of patients’ conditions, the duration of work hours per week, and anxiety	Anxiety and stress	Psychological issues	Psychosocial challenges
2	Sagherian et al., 2020 United States	• Insomnia, fatigue (acute and chronic), low-to-moderate inter-shift recovery, emotional exhaustion, depersonalization, psychological distress (depression and anxiety) and post-traumatic stress disorder. • Contributing factors included being a frontline nurse, and increased working hours per week. These resulted in insomnia, fatigue, low inter-shift recovery, burnout and PTSD). • Rest breaks were identified as means of coping with challenges	Insomnia, fatigue, low-to-moderate inter-shift recovery, emotional exhaustion, depersonalization, psychological distress and post-traumatic stress disorder	Psychological issues	Psychosocial challenges
**3**	Arnetz et al., 2020 United States	Sources of Stress among Nurses • fear of exposure to COVID-19 infection. • fear of the death of patients, co-workers and loved ones. • the feeling of inadequacy and helplessness in caring for COVID-19 patients • scarcity of PPEs and the discomfort related to wearing them • false information sharing on COVID-19	Fear of contracting the disease and exposure to death Helplessness Inadequate PPE supply False information	Fear Psychological issues Inadequate resources Poor information flow	Sources of Stress among Nurses Psychosocial challenges
4	Cai et al., 2020 China	• Key work-related challenges: depression, anxiety, insomnia, and PTSD. • The factors that impacted these challenges included nurses’ unit of work (highest among nurses at COVID-19 units), changes in the physical state of nurses, and doubt about the fight against the pandemic. • Access to online psychological information was valuable and served as a sufficient protection impact factor for anxiety, insomnia, and PTSD symptoms	Depression, anxiety, poor sleep, and PTSD Access to psychological information	Psychological issues Access to information	Psychosocial challenges Coping strategy
5	Coffré & de los Ángeles Leví Aguirre, 2020 Ecuador	• Fear/stress associated with caring, dissatisfaction with working extended hours, and increased turnover intention • Factors relating to frequency, intensity and possibility of transmission of COVID-19 to relatives, getting infected through handling patients, and lack of personal protection equipment. Lack of treatment and vaccines for the virus; television and social media news reportage about COVID-19; observing anxious and frightened colleagues, and having possible symptoms of the disease Coping strategies • Exhibition of a positive attitude; • Assurance had improved COVID-19 cases, with no relations getting infected. • Sticking to the same or even reducing shift hours at work • Safety nursing practice • Strictly following personal protection measures, • Maintaining separate clothing for the street and work. • Acquiring more knowledge about the disease. • Avoiding public places. • Strategic communication with relatives and friends. • Improved nutrition, • Physical exercise and recreation. Expression of feelings.	Stress and extra working hours Fear of the disease and transmitting it to others Psychological approach to coping Adherence to safety protocols Communication Taking good care of oneself	Psychological issues Fear Coping mechanism	Psychosocial challenges Sources of stress Coping
*6*	Galletta et al., 2021 Italy	Work-related challenges included • Fear of putting family at risk of getting infected • The feeling of inadequacy in preparation for the pandemic • High level of rumination about the pandemic **Causes of work stress**: • Watching colleagues crying at work-induced stress among nurses. • Increased job stress • Increased job demand • Worry about getting infected.	Fear of the disease Inadequacy and helplessness Visible helplessness	Fear Psychological and social issues Psychological and social issues	Source of stress Psychosocial challenges Psychosocial challenges
7	Marthoenis et al., 2021 Indonesia	• Most nurses experienced depression, anxiety, stress and social rejection by family and neighbours • Associated risk factors included: ♣ Nature of the work area of the nurse (COVID-19 and non-COVID-19 centres), ♣ Financial hardship due to the pandemic, ♣ Social rejection by family due to nurses’ proximity to COVID-19 patients. ♣ Frequent watching of news on TV about COVID-19. ♣ Persistent use of crowded places. ♣ Feeling worried about the pandemic. Optimism that the government may win against COVID-19 and appropriate behaviour such as wearing a face mask whenever they leave their homes.	Depression, anxiety, stress Social rejection by family	Psychological issues Social issues	Psychological challenges
8	Poortaghi et al., 2021 Iran	• Nurse managers volunteered to support frontline nurses during shortages and work overload. • Introduction of flexible work schedule through rearrangement of the workforce by reassigning high-risk staff, e.g., aged, pregnant and lactating nurses, and nurses with underlying medical conditions to work areas of low risk of COVID-19. • The preventive measures include training on COVID-19, provision of adequate PPEs and allocation of places for quarantining of patients and staff was ensured • Financial incentives • Close communication between staff and managers • Increased off time between shifts	Support from nurse managers Flexible work schedule	Availability of support Flexible work schedule	Coping Coping
*9*	Sadang, 2021 Philippines	• Fear and worry • Increased workload & burnout • Stigmatization • Higher risk of contracting and spreading the virus, • Rise in infected cases • Poor knowledge of patients and the public on COVID-19	Fear and worry Burnout and stress Stigmatization	Psychological issues Psychological issues Social issues	Psychosocial challenges Psychosocial challenges Psychosocial challenges
10	Lorente et al., 2021 Spain	• Fear of infection and death and dying • Higher levels of rumination • Watching co-workers cry at the workplace. • Increased job demand • Lack of appropriate support system at the workplace. • Emotion-focused coping (to improve resilience, insufficient preparation, lack of support, and fear of infection) • Problem-focused coping (stress from work overload).	Fear of infection and death Rumination Emotion-focused coping Problem-focused coping	Fear Psychological issues Coping mechanism Coping mechanism	Source of stress Psychosocial challenges Coping Coping
11	Moradi et al., 2021 Iran	• Lack of organizational support • Physical exhaustion • Uncertainties • Psychological stress	Limited support Stress and exhaustion	Limited support Exhaustion	Source of stress
12	Xiong et al., 2020 China	• Anxiety • Depression The self-efficacy coping • Psychological assistance intervention o Guiding Principles for psychological intervention during COVID-19 o Psychological guidelines books o Psychological assistance hotlines Online psychological counselling	Anxiety and depression Psychological support	Psychological issues Support	Psychosocial challenges Coping
13	L. Zhang et al., 2021 China	• Burnout • High level of emotional exhaustion • High level of depersonalization • Low level of personal accomplishment • Mental health guidance • Stress coping techniques	Exhaustion and burnout Psychological support	Psychological issues Coping mechanism	Psychological challenges Coping
14	Zheng et al., 2021 China	• Depression • Anxiety • Stress	Anxiety, depression, and stress	Psychological issues	Psychosocial challenges
15	Waage et al., 2021 Norway	• Change in sleep duration • Poor sleep quality	Altered sleep	Psychological issues	Psychosocial challenges
16	X. Zhang et al., 2021 China	• Burnout • Psychological distress • Posttraumatic stress Adaptive coping	Burnout and distress Adaptive coping	Psychological issues Coping mechanism	Psychosocial challenges Coping
17	Zhan et al., 2020 China	• Job stress	Stress	Stress from the job	Sources of stress
18	McFadden et al., 2021, United Kingdom	• Lower levels of well-being and quality of working life • Positive coping strategies (e.g., active coping, positive reframing, acceptance) and negative coping strategies (e.g., venting, behavioural disengagement, self-blame) were used	Well-being and quality of working life Positive coping and negative coping	Psychological issues Coping strategies	Psychosocial challenges Coping
19	Nowell et al., 2021 Canada	• Nurses lacked confidence and experienced a state of chaos and anxiety • Workplace factors including the adequacy of personal protective equipment, clear information and guidance, supportive leadership, teamwork and adequate staffing influenced nurses’ confidence.	Lack of confidence, chaos and anxiety Personal protective equipment, clear information, guidance, supportive leadership, teamwork, adequate staffing	Psychosocial issues Coping strategies	Psychosocial challenges Coping
20	Fteropoulli et al., 2021 United Kingdom	• Prevalence of moderate to severe anxiety and clinically significant depression. • Depression and occupational burnout were significant risk factors for poor quality of life. • A significant risk factor for poor psychological outcomes was perceptions of inadequate workplace preparation to deal with the pandemic • Approach (active efforts to deal with the problem), Support-seeking (seeking support from the environment), and Avoidance (avoiding dealing with the problem) coping were strategies used to overcome the psychosocial impacts of the COVID-19 pandemic.	Anxiety, depression, occupational burnout, poor quality of life Inadequate workplace preparation Approach, support-seeking and avoidance	Psychological outcomes Coping strategies	Psychological challenges Coping
21	AlJhani et al., 2021 Saudi Arabia	• Burnout was higher among nurses. • Adaptive (religion, acceptance, active coping, planning and positive reframing and maladaptive coping (self-distraction, venting and denial were used by nurses as coping strategies.	Burnout Adaptive coping, maladaptive coping	Psychological issues Coping strategies	Psychological challenges Coping
22	Molero-Jurado et al., 2021 Spain	Coping strategies such as rumination, self-blame, blaming others, positive refocusing, positive reappraisal and acceptance were related to the presence of health problems (presence of anxiety/insomnia, social dysfunction, and depression).	Rumination, self-blame, blaming others, positive refocusing, positive reappraisal and acceptance	Coping strategies	Coping
23	Cui et al., 2021 China	• Anxiety symptoms and stress were high among nurses • Positive professional attitudes and being trained in emergency preparedness cope well with anxiety and stress	Anxiety and stress Positive professional attitude, emergency preparedness	Psychological issues Coping strategies	Psychological challenges Coping
24	Alameddine et al., 2021 Lebanon	• Burnout (personal, work-related and client-related) was low and moderate among most of the nurses • Resilience was associated with burnout; burnout tends to reduce resilience and vice versa	Burnout Resilience	Psychological issues Resilience	Psychological challenges Coping
25	Htay et al., 2021 Multi-countries	Among the common coping strategies used during the COVID-19 pandemic included getting family support, positive thinking, prayers and worshipping according to one’s beliefs and adequate sleep and food intake.	Family support, positive thinking, religious activities, recreation, staying away from fake news, sleep and a good diet	Coping strategies	Coping
26	Kotrotsiou et al., 2021 Greece	Positive approach, search for social support, wishful thinking, avoidance and problem-solving assertion strategies correlated significantly with the socio-demographic characteristics of nurses.	Positive approach, social support, wishful thinking, avoidance, problem-solving assertion	Stress management strategies	Coping
27	Engelbrecht et al., 2021 South Africa	• Nurses had higher levels of PTSD • Approach coping (acceptance, use of instrumental support, use of emotional support, positive reframing, religion, planning and active coping), avoidant coping (self-distraction, denial, venting, substance use, behavioural engagement and self-blame), humour and religion were some of the strategies used to cope with the PTSD.	Post-traumatic stress disorder Acceptance, support, reframing, planning, self-distraction, denial, venting, substance use, engagement, self-blame, humour, religion	Psychological issue Coping strategies	Psychological challenge Coping
28	Hummel et al., 2021 Parts of Europe	• Different levels of depression, anxiety, and stress for each of the 8 European countries. • The causes included uncertainty about when the epidemic will be under control, worry about inflicting COVID-19 on family, worry about nosocomial spread, frequent modification of infection control procedures, conflicts at work as the equivocal definition of responsibility between doctors and nurses and blame from commanding • Among the strategies used in coping included taking protective measures (washing hands, wearing a mask, taking own temperature, etc.), actively acquiring more knowledge about COVID-19 (symptoms, transmission pathway, etc.), video-chatting with family and friends by phone to share concerns and support, engaging in recreational activities (online shopping, social media, internet surfing, etc.), engaging in health-promoting behaviours (more rest, exercise, balanced diet, etc.) and switching thoughts and facing the situations with a positive attitude	Depression, anxiety, stress Uncertainty, family worry, infection, conflicts Protective measures, more knowledge, video chatting, recreation, health promotion, positive attitude	Psychological issues Work-related challenges Coping strategies	Psychological challenges Coping
29	Norman et al., 2021 United States	• Moral distress was high among participants and was associated with PTSD symptoms, burnout, and work and interpersonal functional difficulties • Worries about infecting family, not being able to visit or assist loved ones who had become ill, and not being able to do enough for COVID-19 patients were factors that contributed to moral distress.	Distress, PTSD, burnout, functional difficulties	Psychosocial issues	Psychosocial challenges
30	Ali et al., 2020 United States	• Increased stress, burnout, anxiety, depression and fatigue among frontline nursing staff • The stress of nursing staff was related to taking care of COVID-19 patients, assignments and workload, worry from personal life, friends and colleagues, lack of knowledge about COVID-19, and the work environment • Avoidance, problem-solving, transference, spending time with children, the use of arts and crafts and drinking alcohol were some of the coping strategies used.	Stress, burnout, anxiety, depression, fatigue Avoidance, problem-solving, transference, time with children, alcohol	Psychosocial issues Coping strategies	Psychosocial challenges Coping
31	Norful et al., 2021 United States	• Fear of uncertainty and physical and psychological manifestations of stress were common in nurses • Shifting information, a lack of PPE, and fear of infecting others were the causes of worry for nurses. • Resilience building through organizational efforts, individualized stress mitigation, social support, social media and organizational transparency were reported to be effective against rising stressors.	Fear, uncertainty, stress Resilience, stress mitigation, social support	Psychological issues Coping strategies	Psychological challenges Coping
32	Jerg-Bretzke et al., 2021 Germany	• Stress was high among nurses during the COVID-19 pandemic • The greatest sources of stress included fear of a patient dying, fear of infecting loved ones and family, physical or mental exhaustion and change in tasks.	Stress Fear of dying, mental exhaustion, changes in task	Psychological issues	Psychological challenges
33	Said and El-Shafei, 2020 Egypt	• Occupational stress is higher among frontline nurses Workload, dealing with death and dying, inadequate emotional preparation, problems relating to supervisors and peers, discrimination, conflicts with physicians, uncertainty concerning treatment, and patients and their families were the major causes of stress among nurses.	Occupational stress Workload, death and dying, emotional preparation, the problem with people, discrimination, conflicts, uncertainty	Psychological issues	Psychological challenges
34	Alameddine et al., 2021 Lebanon	Most nurses had moderate resilience. Personal competence, high standards, tenacity, trust in one’s instinct, tolerance of negative effects, strengthening effects of stress, positive acceptance of change and secure relationships, control and spirituality improved the resilience of nurses.	Resilience, competence, high standards, tenacity, tolerance, positive acceptance, control, spirituality	Resilience	Coping
35	Hamama et al., 2021 Israel	• Job stress and psychological distress were reported among nurses. • Causes of stress included inadequate PPE at their workplace, little information on how to manage safety workplace and inadequate attention by organizations to the needs arising from the COVID-19 outbreak	Job stress, distress Inadequate resources, lack of information, limited attention	Psychological issues	Psychological challenges
36	Kowalczuk et al., 2021 Poland	• Excessive sleepiness and insomnia were identified among nurses • Coping strategies include active strategies (active coping, planning and positive reframing), support-seeking and emotion-oriented strategies (religion, use of emotional support, use of instrumental support, venting and self-blame) and avoidant strategies (acceptance, behavioural disengagement, denial, self-distraction, substance use, humour, religion) were used to advance in overcoming sleep problems.	Sleep disorders Active coping, support-seeking, avoidance	Psychological issues Coping strategies	Psychological challenges Coping
37	Khatatbeh et al., 2021 Jordan	• Nurses experienced emotional reactions (feelings of fear, worries, and anxiety), social stigma, extreme workload • Factors that increased the fear, stress, and anxiety included having an old aged parent with co-morbidity in the home, worry of spreading the infection to family and inadequate knowledge and unclear situation of the COVID-19	Fear, worry, anxiety, stigma, increased workload	Psychosocial issues	Psychosocial challenges
38	Hong et al., 2021 South Korea	• Nurses were rated as having clinical depression and also presented anxiety, insomnia and work-stress	Depression, anxiety, insomnia and work-stress	Psychological issues	Psychological challenges
39	Lee et al., 2021 South Korea	• Nurses experienced work-related stress in the form of depression, anxiety, or insomnia. • Coping behaviours included having conversations with people, partaking in hobbies and exercise, partaking in behaviours such as smoking and drinking and using social network services (SNS) via the internet	Depression, anxiety, insomnia, stress Conservation, hobbies, exercise, smoking, drinking alcohol, social media usage	Psychological issues Coping strategies	Psychological challenges Coping
40	Betke et al., 2021 Poland	Problem-focused strategies (active coping, planning and positive reframing) were used by nurses to deal with stress.	Planning, active coping, reframing	Coping strategies	Coping
41	Chui et al., 2021 Malaysia	• There was stress and depression among nurses • Highly stressed or depressed nurses tend to adopt avoidance (self-blame, venting and substance use), religion, reframing, active coping, planning and emotional support were used regardless of the stress or depression levels experienced.	Stress and depression Avoidance, religion, emotional support, planning, substance use, self-blame, venting, reframing	Psychological issues Coping strategies	Psychological challenges Coping
42	Aboutiq and Borchardt, 2021 United States	• Occupational stress was high • The top occupational stressors included wearing a face mask at all times in the hospital, unpredictable staffing and scheduling, not enough staff to adequately cover the unit, feeling helpless in the case a patient fails to improve, and being assigned to a COVID-19 patient.	Stress Wearing a face mask, under-staffing, scheduling, helplessness, assigning to COVID-19 patient	Psychological issues	Psychological challenges
43	Marcolongo et al., 2021 Italy	• The study showed nurses exhibiting anxiety, depression and fear caused by the COVID-19 pandemic. Low resilience was also recorded. • Acceptance, planning, active coping, instrumental and emotional support, and self-distraction are the most used strategies by health workers.	Anxiety, depression, fear and low resilience Active coping, avoidance, emotional support	Psychological issues Coping strategies	Psychological challenges Coping
44	McFadden et al., 2021 United Kingdom	• Low quality of working life • The most frequently used coping strategy was acceptance, behavioural disengagement, family–work segmentation	Quality of work-life Acceptance, disengagement, family-work segmentation	Psychosocial issue Coping strategies	Psychosocial challenges Coping
45	Labrague and de los Santos, 2020 Philippines	Fear of COVID-19 and psychological distress were high among nurses	Fear, psychological distress	Psychological issues	Psychological challenges

### Psychological challenges

The psychological problems came in the form of a higher level of burnout, higher levels of depression, intense anxiety, post-traumatic stress disorder (PTSD), sleep disorders, low quality of life, and fear of infection and death. These problems arose because nurses saw a lot of people dying including their colleagues and loved ones. They felt hopeless, helpless, and inadequate in their caregiving roles. In addition, false information gotten from social media and television made nurses uncertain about the viral dynamics because of the different variants that kept coming up (18, 20, 22, 23, 25–27, 29–32, 34, 35, 37–45, 47–57, 59, 60, 62–64).

### Social challenges

Front-line nurses were separated from their families because of the virulent nature of the virus. In addition to the various lockdowns that were imposed on countries with higher case fatalities and infection rates also affected them. Business closures affected nurses with financial insecurities. Nurses had uncertainty about their job and work output (30, 42, 62).

### Factors accounting for work-related psycho-social Challenges among nurses

In the present review, immense challenges were identified in the work environment of nurses which contributed to their psychosocial problems. These factors include the inadequate supply of resources and uneasiness associated with the use of personal protective equipment (PPEs), lack of fixed guidelines on case management, infection prevention protocols, and low inter-shift recovery. The inadequate PPEs supply put nurses at higher risk of contracting the virus [18, 21, 23, 28–31, 49, 59, 60, 62].

Other factors included the severity of patients’ conditions and working in COVID-19 centres as a frontline nurse. Frontline nurses were worse in depression, anxiety, and stress levels than non-frontline nurses. Increased workload and hours per week, and the tedious shift system contributed to insomnia, fatigue, low inter-shift recovery, burnout, and PTSD, though the frequency of 30-minute breaks was significant in reducing some of the challenges. The stress level of nurses was attributed to fear of exposure to infections from COVID-19, and fear of illness/death of the patient, co-workers, and/or loved ones. Additionally, nurses felt inadequate and helpless in the care of COVID-19 patients at the workplace, especially in the event of the high incidence of false information about COVID-19 in the media [23, 30, 34, 35, 40, 45, 47, 48, 56, 57, 62]. Cai et al. [56] and Coffré and Aguirre [28] on the other hand indicated that watching colleagues cry at work, induced stress among nurses, and was associated with the age of the nurse, as younger nurses were more worried about the health of their families, patients, and colleagues compared to experienced nurses. Similarly, being single or divorced, the female gender and the position of nurses in their families (being the only child in their families) influenced the work-related psychosocial challenges. Higher levels of contemplation resulted from increased job stress and increased job demand. The lack of specific treatment for COVID-19, vaccines unavailable, financial hardship, social rejection, and stigmatization of nurses, all contributed to various work-related challenges [30, 42, 48, 51, 52].

### Coping strategies

The strategies used to resolve work-related challenges were either nurse-specific or an institutional-established plan to cope with the challenges of the pandemic. First, nurses used emotion-focused coping (EFC) or problem-focused coping (PFC) such as avoidance, religion, emotional support, planning, active coping, substance use, self-blame, venting, and reframing depending on the nature of the challenge. While EFC was used to improve the resilience of nurses (lack of support, insufficient preparation, and fear of infection), PFC was more helpful in dealing with psychological stress (fear of infection and work overload). Most nurses, however, combined both coping strategies [19, 25, 27–29, 33, 37, 40, 44, 46, 51, 54]. The exhibition of a positive attitude among nursing team members and assurance of nurses that COVID-19 cases improve was also helpful in dealing with the challenges.

Accessing online psychological information had a protective impact factor for anxiety, insomnia, and PTSD symptoms [51, 56, 64]. Most nurses reiterated that sticking to the same or even reduced hours in a shift at work or a flexible work schedule helped them cope with the stresses [59, 61]. Coping strategies related to the safety of nursing practice: strictly following personal protective measures, for example, maintaining separate clothing for the street and work, constant use of masks, and COVID-19 knowledge acquisition were listed as activities undertaken. Other measures included effective communication with relatives and friends; positive thinking and attitudes, and improved nutrition, exercise, and recreational activities were helpful coping measures [22, 25, 28, 34, 39].

Rest breaks, daily self-health monitoring, vaccination, and the use of anti-viral sprays and thymus injections to enhance immunity were also effective for most nurses in COVID-19 centres [65]. Online counselling, advice hotlines, and online chat rooms for frontline nursing staff were explored in handling psychological distress. Mindfulness-based intervention is aimed at reducing stress through mindful meditation practices [26, 28, 36, 39, 40, 44, 56]. Management-related strategies designed by facilities to support nurses with work-related challenges: continuous guidance and psychological assistance by management, training schedules for staff including orientation to general ward work and nursing responsibilities, infection control and self-protection, mental health guidance to orient younger and less experienced nurses during the pandemic response and provision of sufficient work resources including PPEs, benefits such as financial and non-financial incentives and promotions packages to engage in volunteer workforces to support frontline during shortage and work overload [59, 61]. Others included the introduction of flexible work schedules through rearrangement of the workforce, thus relocating high-risk staff, e.g., aged, pregnant, and lactating nurses and nurses with underlying medical conditions to work areas of low-risk of COVID-19 [24, 25, 28, 34, 37, 46, 48, 61].

## Discussion

It is essential to synthesize evidence to improve practice and inform policy in nursing care and workforce management. This review identified studies that aimed to determine work-related psychosocial challenges nurses faced while caring for COVID-19 patients and summarised the most common work-related psychosocial challenges, factors accounting for the challenges, and coping strategies nurses use in dealing with them across the globe. The impact of the pandemic, which places in perspective the challenges on healthcare facilities and the nursing workforce is enormous [66, 67].

The commonest psychosocial work-related challenges included stress, anxiety, depression, sleep difficulties, burnout, PTSD, fear of infection and death, and stigmatization. These work-related psychosocial stresses resulting from COVID-19 are likely to have more repercussions on nurses even after the pandemic [68] as past episodes of pandemics have demonstrated similar findings. According to Xiao et al. [69], nurses who provided care during SARS experienced stress and psychological discomfort which accounted for 68% and 57%, respectively. Likewise, between 29 and 35% of nurses reported experiencing a significant level of distress, and the pertinent contextual characteristics were working with SARS patients, being a nurse, and being a parent.

The death of people including nurses is alarmingly causing a lot of fear, depression, and anxiety [5, 70, 71]. Aside from the fear of death, anxiety, depression, and PTSD, nurses also experience burnout due to excessive workload and psychological distress. Likewise, stigmatization has resulted from working as a frontline in a stressful environment [72]. Similar findings have been reported in the past by Bernaldo-De-Quirós et al. [73] and Donnelly and Siebert [74] while occupation and other sociodemographic factors are major contributors to burnout [75, 76]. COVID-19 has been a major significant challenge confronting nurses and, therefore advocacy to better the psychological well-being of nurses is essential through the various waves of the pandemic.

Coping strategies were aimed at insulating nurses from both physical and psychological challenges. Though rest break has been mandatory in most public organizations, their impact has not been adequately reported concerning nursing care outcomes. In the past, most nurses did not consider the significance of rest breaks as a means of coping with the challenges of the stressful work environment. Findings from the current review demonstrated the need to institute compulsory rest breaks in the daily schedule of the nurse as physical restitution, decreased feeling of being sick, improved safety-related decision-making, and the general well-being of nurses is associated with rest breaks during work [77, 78]. Again, the usefulness of problem-focused and emotion-focused coping strategies in reducing the psychological burden on nurses has been explored in the past. Emotion-focused coping has been demonstrated to be effective when dealing with emotional trauma associated with work. Nurse managers responsible for policymaking in the healthcare system should consider developing culturally sensitive coping strategies. Resilience among nurses can be empowered by integrating coping strategies in the educational and orientation programs for nurses [19, 20].

Though much cannot be said about the use of online social support such as chat rooms, counselling and advice by healthcare providers facing work-related stresses, such coping strategies have been used to provide support [79, 80]. The opportunity created through these media enhanced the ability of nurses to share their emotions (sadness and fear). These findings are consistent with other studies that saw a positive association between the use of technological support and the reduction of fear and depression among the health workforce [81–83]. The role of online advice, chat rooms, and counselling in reducing psychological stress among nurses is promising and calls for further studies on it.

Meanwhile, major difficulties encountered during the pandemic were increased workload and the shortage of PPEs which is similar to reported cases of health commodity shortages during the Ebola outbreak [84, 85]. Due to the highly contagious nature of the COVID-19 pandemic, all facets of the health system including health financing and human resource have been challenged, and the effect is global shortages of PPEs. Safeguarding the judicious supply of quality and quantity health commodities is germane to the emergency response systems globally. Measures at increasing the training of such cadres of the nursing workforce will help reduce the workload on these few specialist nurses, thereby improving efficiency in the provision of delicate but complex nursing care to patients. Implementing a scientifically arranged shift system through rational allocation of the nursing workforce and flexible duty roster can reduce the physical and psychological challenges associated with the pandemic [86].

The subject has been a great concern in managing human resources in health services, and therefore it is essential to fully appreciate and ease its effects on patients and workforce safety. This position is supported by Ramaci et al. [87] and Schubert et al. [88] who reiterate that supportive strategies against stigma are recommended at workplaces to promote nurses’ well-being, as stigmatization can impact nurses’ work ethics in the management of COVID-19 patients. Policy measures on the work environment are, therefore, needed to ensure the motivation and job satisfaction of nurses. This will go a long to reducing turnover associated with the COVID-19 pandemic [89].

Challenges of physical and psychological stress are resolved through a flexible shift system for experienced and inexperienced nurses. The support system at work from nurse managers also contributes immensely to the coping of nurses [90, 91]; these are similar to findings from the review. This review supports findings of management-instituted support through building a strong nursing workforce teamwork, provision of psychological counselling units for nurses, incentives packages, and other external support for nurses during the pandemic [2, 92, 93]. These strategies help motivates nurses, and also reduce the infection- and death-induced fear associated with the provision of care to patients. As a way of building resilience, developing an optimistic attitude towards life is helpful in psychosocial work-related challenges, and enhances the overall well-being of the workforce. Again, our review demonstrated the establishment of programmes designed to educate nurses on COVID-19 and also on how to deal with stress at work through emotion-focused and problem-focused coping, and this was recommended in past studies [94–96].

Furthermore, while some nurses experienced social isolation due to being separated from loved ones and friends, others experience stigmatization. Congruent with other studies, nurses also go through the ethical dilemma and are found battling with either quitting their job as their families mount pressure on them to quit the job due to stigma or fulfilling professional goals and values [71, 97]. This calls for the attention of all stakeholders to find a lasting solution to these problems endangering the lives of nurses across the globe.

## Conclusion and implications

The final stage of the scoping review dealt with the conclusion of the findings. The studies reviewed were mostly from China, with a few from other parts of the world. Meanwhile, the literature indicates that Africa, as a resource-constrained continent, will have serious consequences from COVID-19 for the healthcare workforce, especially the nursing workforce. However, the authors came across only one primary study done in Africa specifically on nurses’ work-related challenges as frontline health workers. We, therefore, recommend that more studies concentrated on African nurses so that their unique concerns can be brought to bear. Again, 39 out of the 45 studies reviewed were quantitative studies, and six qualitative studies were reviewed. We also recommend more qualitative studies be done to gain a deeper understanding of the challenges nurses faced from their perspectives. Finally, it was observed that most of the psychological challenges were more pronounced in the female gender, which is also predominant in the nursing profession. Policy decisions could be made to increase the intake of male nurses, who seem to be more resilient and can withstand pressure to provide support in fluid situations like the current pandemic. In the future and subsequent waves, nurse managers should select experienced staff who are not too old and can withstand the virulence of the virus. This study’s findings are not surprising because of the nature of the pandemic, the dominance of the female gender in the nursing profession, and the roles of the female gender in homes and society. What is worrying therefore is the fact that nurses are dying, and some have depression and PTSD. This invariably is going to affect the nursing profession, the overall quality of life of the nurses, and ultimately, the realisation of UHC and SDGs. The WHO, health institutions/hospitals, and nursing organisations both international and local should plan activities to give universal psychotherapy to nurses across the globe to help them recover fully from the effects of the pandemic. It is also recommended that healthcare facilities prioritize the following steps in the event of a future pandemic institute more psychological support systems at the workplace for nurses and also enhance coping skill training. Again, to create a safe environment to minimise the spread of infectious disease in hospitals, suitable medical protective equipment must be installed, and there must be a commitment from all stakeholders to building resilient healthcare to withstand any unforeseen events in the future. This would foster a positive atmosphere and ensure the nurses’ safety, enabling them to continue providing the highest standard of patient care to defeat this disease.

### Limitations

Despite the interesting findings obtained, some limitations are noteworthy. Firstly, only studies with a full article were considered for inclusion, which led to the exclusion of studies with only the abstract. Secondly, the quality of the included primary studies was not assessed due to the scoping nature of the study. Thus, the findings may be interpreted with caution. Again, a critical appraisal of the sources of evidence was not conducted and, as such, was not reported.

## Data Availability

All data generated or analyzed during this study are included in this published article. Any other data are also available from the corresponding author on reasonable request.

## Abbreviations

COVID-19: Coronavirus disease
EFC: Emotion-focused coping
ICU: Intensive Care Unit
PFC: Problem-focused coping
PPE: Personal Protective Equipment
PTSD: Post-Traumatic Stress Disorders
SARS-COV-2: Severe Acute Respiratory Coronavirus 2
SDGs: Sustainable Development Goals
UHC: Universal Health Coverage
WHO: World Health Organization
